# Vitamin A biomarkers were associated with *α*(1)-acid glycoprotein and C-reactive protein over the course of a human norovirus challenge infection

**DOI:** 10.1017/S0007114523002076

**Published:** 2024-02-14

**Authors:** Courtney P. Victor, Juan S. Leon, Anne M. Williams

**Affiliations:** 1 Hubert Department of Global Health, Emory University, Atlanta, GA 30322, USA; 2 Department of Human Nutrition, University of Otago, Dunedin, New Zealand

**Keywords:** Retinol, Vitamin A, Retinol binding protein, Inflammation

## Abstract

Retinol binding protein (RBP) is used as a proxy for retinol in population-based assessments of vitamin A deficiency (VAD) for cost-effectiveness and feasibility. When the cut-off of < 0·7 μmol/l for retinol is applied to RBP to define VAD, an equivalence of the two biomarkers is assumed. Evidence suggests that the relationship between retinol and RBP is not 1:1, particularly in populations with a high burden of infection or inflammation. The goal of this analysis was to longitudinally evaluate the retinol:RBP ratio over 1 month of follow-up among fifty-two individuals exposed to norovirus (*n* 26 infected, *n* 26 uninfected), test whether inflammation (measured as *α*-1-acid glycoprotein (AGP) and C-reactive protein (CRP)) affects retinol, RBP and the ratio between the two and assess whether adjusting vitamin A biomarkers for AGP or CRP improves the equivalence of retinol and RBP. We found that the median molar ratio between retinol and RBP was the same among infected (0·68) and uninfected (0·68) individuals. AGP was associated with the ratio and RBP individually, controlling for CRP, and CRP was associated with both retinol and RBP individually, controlling for AGP over 1 month of follow-up. Adjusting for inflammation led to a slight increase in the ratio among infected individuals (0·71) but remained significantly different from the expected value of one. These findings highlight the need for updated recommendations from the WHO on a cut-off value for RBP and an appropriate method for measuring and adjusting for inflammation when using RBP in population assessments of VAD.

The WHO recommends the use of serum retinol (i.e. serum vitamin A) as a biomarker for population-level surveillance and evaluation of vitamin A deficiency (VAD)^(1)^. The WHO cut-off for determining VAD is a concentration of <0·7 μmol/l serum retinol; the public health problem is considered severe if greater than 20 % of a population have VAD based on aforementioned cut-off^(1)^. A prevalence of VAD greater than 10 % indicates a moderate public health problem. However, measuring serum retinol for population assessment can be expensive and difficult to complete due to the requirement of a trained phlebotomist for sample collection, cold-chain storage, lab equipment and expertise for HPLC analysis^(2)^.

Retinol binding protein (RBP), a serum carrier protein for retinol, is a negative acute phase protein. It is synthesised in the liver and is steadily released from the liver into the blood bound to serum retinol and transthyretin in a 1:1:1 molar complex in order to meet daily tissue needs for vitamin A^(3,4)^. RBP is often used as a proxy for retinol in population-based assessments of VAD because analytic assessment of RBP is simpler and more cost-effective: ELISA requires less technical expertise and equipment than HPLC analysis. Because of the physiology of retinol and RBP, they are assumed to be equimolar in the blood serum, and therefore, measuring RBP is a direct (1:1) measurement of serum retinol concentrations^(2)^. There are no WHO recommendations for the use of RBP to define VAD, although a 1:1 molar ratio of retinol to RBP has been expected^(5)^, and the cut-off for serum retinol (<0·7 μmol/l) is often substituted for serum RBP (<0·7 μmol/l) to signify deficiency^(4,6)^. However, research shows the molar ratio between retinol and RBP is inconsistent and can have a substantial (range 0·91–1·52)^(5,7–11)^. A recommended approach for using RBP in population-based studies is to measure serum retinol concentrations from a subset of the population of interest and use these data to predict serum retinol from RBP^(2)^.

One explanation for the inconsistent ratio of retinol to RBP may be that infection and inflammation differentially influence the two biomarkers^(1,5)^. In order to accurately determine vitamin A status in a population, inflammation should be accounted for in biomarker measurements, as it has been demonstrated that inflammation can alter biomarker measurements for several micronutrients in a way that does not reflect the true micronutrient status of the individual^(2)^. A group of researchers developed the Biomarkers Reflecting Inflammation and Nutritional Determinants of Anemia (BRINDA) project. The goals of this project were to assess a number of questions including whether inflammation adjustment was necessary, the necessity of using both CRP and AGP (*α*(1)-acid glycoprotein), both acute phase proteins, in adjustment, and the best method for adjusting for inflammation in micronutrient biomarker measurements^(12)^. A standardised protocol was developed to correct biomarker measurement based on CRP and AGP levels; this approach has been widely adopted by the scientific community in this field in its use for adjustment of ferritin measurements. The BRINDA approach has been recommended by WHO for adjustment of ferritin, and the most recent update by WHO on vitamin A biomarkers was published in 1996^(13)^. Since 1996, the BRINDA project recommends correction for inflammation in vitamin A biomarkers, retinol and RBP, among women and pre-school-aged children. However, some more recent research supports only adjusting for inflammation among women but not pre-school aged children^(14)^. Other approaches for inflammation adjustment include the Thurnham approach, which categorises individuals and applies correction factors based on status: reference (no inflammation), incubation, early convalescence and late convalescence^(15)^. Updated recommendations from the WHO on vitamin A biomarker assessment based on present research are needed.

To quantify the molar ratio of retinol to RBP^(16,17)^ and analyse the impact of infection and inflammation, we used clinical specimens from a human challenge study where fifty-two apparently healthy adults were exposed to norovirus, the most common cause of epidemic gastroenteritis. Our primary objectives were to (1) evaluate the ratio between retinol and RBP among infected and uninfected individuals over the course of a norovirus infection within the human challenge study; (2) assess whether inflammation may alter retinol, RBP and the expected (unadjusted) 1:1 molar ratio between these two biomarkers; and (3) assess whether adjusting retinol and RBP for inflammation alters the molar ratio between retinol and RBP.

## Methods

### Study population and exposure

This research was a secondary analysis of a dataset derived from a human norovirus challenge study conducted in Atlanta, GA, USA in 2006 and 2009^(18,19)^. Serum samples were collected from fifty-two participants enrolled in two separate norovirus challenge studies. Enrolled healthy adults were of positive secretor status, a marker of susceptibility to Norwalk virus, a norovirus strain, infection. In the first study, participants drank 10 ml of water inoculated with Norwalk virus (final dose of 6·5 × 10^8^ GEC of virus)^(19)^. Participants in the second study were inoculated with 1·0 × 10^4^ GEC of norovirus from spiked oysters^(18)^. In both studies, patients were hospitalised for 4 d post-inoculation, released and then returned for weekly follow-up visits for up to 5 weeks post-exposure. From these two studies, twenty-six individuals became infected, as determined by detection of Norwalk virus RNA in stool samples, from a reverse transcriptase polymerase chain reaction at any day post-inoculation. Infected individuals were age-matched with twenty-six individuals from either study who did not become infected and the unique matched pairs were combined into a single dataset. Age-matching of infected participants was prioritised to match ages of uninfected participants within the same (first or second) study, when possible. When age-matching was not possible, infected participants were paired with uninfected individuals from the other study^(20)^.

### Inflammation and vitamin A biomarkers

Serum samples were collected from all participants at baseline (pre-inoculation, day 0) and on days 1, 2, 3 and 4 post-inoculation. Samples were also collected at weekly follow-up visits up to day 35, for a maximum of ten samples per participant. These samples were analysed for nutritional biomarkers to investigate the relationship between inflammation and micronutrient concentrations^(21)^. Serum samples were stored at −80°C from time of extraction (2006 or 2009) to dry ice shipment for analysis in 2016. Samples were quantified for C-reactive protein (CRP, mg/l) and AGP, g/l, retinol and RBP. Serum CRP, AGP and RBP were measured by the VitMin Laboratory in Germany using an antibody-based capture sandwich ELISA and used calibration curves derived from a commercially available control sample^(22)^. Retinol was measured using high-performance liquid chromatography with a UV detector at the University of Madison, Wisconsin. Ratio variables were created by dividing retinol concentrations (μmol/l) by RBP concentrations (μmol/l) at each time point per subject.

### Statistical analyses

All statistical analyses were conducted in R statistical software (RStudio v. 1.3.1093). To assess baseline differences in demographic variables between the infected and uninfected individuals, we used Wilcoxon signed-rank tests. There were fifty-two individuals initially selected for this study. One individual was excluded due to missing baseline data. Of the fifty-one remaining participants, twenty-five individuals became infected and twenty-six remained uninfected. Retinol data were missing for six other individuals at various (0–4, 21, and 25) time points throughout the study. Inflammation data were missing for six of the infected individuals in the study population. There were a total of eight missing person-time observations from the final dataset (*n* 502).

(1) Our first objective was to evaluate whether the ratio between retinol and RBP was 1:1 in infected *v*. uninfected individuals over the course of a norovirus infection. We assessed this using one-sample Wilcoxon tests comparing the median pooled ratio among infected and uninfected individuals separately to the expected value for the ratio of one.

(2) The second objective was to assess whether inflammation was associated with retinol, RBP and the unadjusted molar ratio between retinol and RBP over the course of a norovirus infection. We used mixed effect models with a random intercept for the study subject to assess the relationship between AGP and CRP with retinol, RBP and the unadjusted molar ratio of retinol to RBP over the course of the study, controlling for day post-infection. In order to evaluate the temporal associations between each inflammatory biomarker (CRP and AGP) and the vitamin A biomarkers (retinol, RBP and the molar ratio of retinol to RBP), we created unique linear models of these relationships by day. These ten models per vitamin A biomarker enabled elucidation of patterns across the time series following the challenge infection (e.g. during the acute phase response *v*. once subjects had recovered from infection). Confounding by both inflammatory biomarkers was assessed and included in the final model where appropriate.

(3) The final objective was to assess whether adjusting the vitamin A biomarker measurements for inflammation would impact the molar ratio between retinol and RBP. To adjust retinol and RBP for the influence of inflammation using the BRINDA approach^(23)^, linear regression was used to subtract the influence of CRP and AGP on each nutritional biomarker using the BRINDA R package^(12)^. A new retinol:RBP ratio variable was created using BRINDA-adjusted measures for retinol and RBP. The unadjusted and inflammation-adjusted ratio variables were pooled across study participants by day post-inoculation and visually compared with the expected ratio of 1. Statistical significance was defined by an *α* level of 0·05. Daily ratio variables stratified by infection status were compared with expected ratio of 1 using one-sample Wilcoxon tests. A paired Wilcoxon test assessed daily significant differences in the unadjusted *v*. adjusted ratios among infected individuals. Outliers were classified as any value greater than 1·5 times the interquartile range of the ratio variable.

## Results

The objectives of this study were to (1) evaluate the ratio between retinol and RBP over the course of an infection within the human challenge study; (2) assess whether inflammation may alter retinol, RBP or the expected (unadjusted) 1:1 molar ratio between these two biomarkers; and (3) assess whether adjusting for inflammation alters the molar ratio between retinol and RBP. There were no statistically significant baseline differences, between infected and uninfected populations, that may impact the study results in age, sex or baseline concentration of retinol, RBP, CRP or AGP (Table 1).

**Table 1.** Baseline characteristics of study population (*n* 51) of adults exposed to a norovirus immunologic challenge

	Infected (*n* 25)		Uninfected (*n* 26)		*P*-value*
	*n*	%	*n*	%	
Male	10	40	12	46	0·87
	Median	Min, Max	Median	Min, Max	
Age	25	19, 51	25	19, 50	0·89
Retinol (μmol/l)†	1·31	0·83, 1·85	1·27	0·81, 2·29	0·76
RBP (μmol/l)	2·00	1·13, 3·04	1·74	1·07, 3·07	0·17
CRP (mg/l)	0·79	0·10, 10·90	1·04	0·03, 10·9	0·89
AGP (g/l)	0·56	0·31, 0·98	0·64	0·27, 1·15	0·34

* Wilcoxon signed-rank tests were run to assess for significant differences in continuous and categorical baseline study characteristics, respectively.

† Retinol data were missing for 1 of the infected individuals at baseline (4 %) and for 1 uninfected individual at baseline (4 %).

**Fig. 1.**
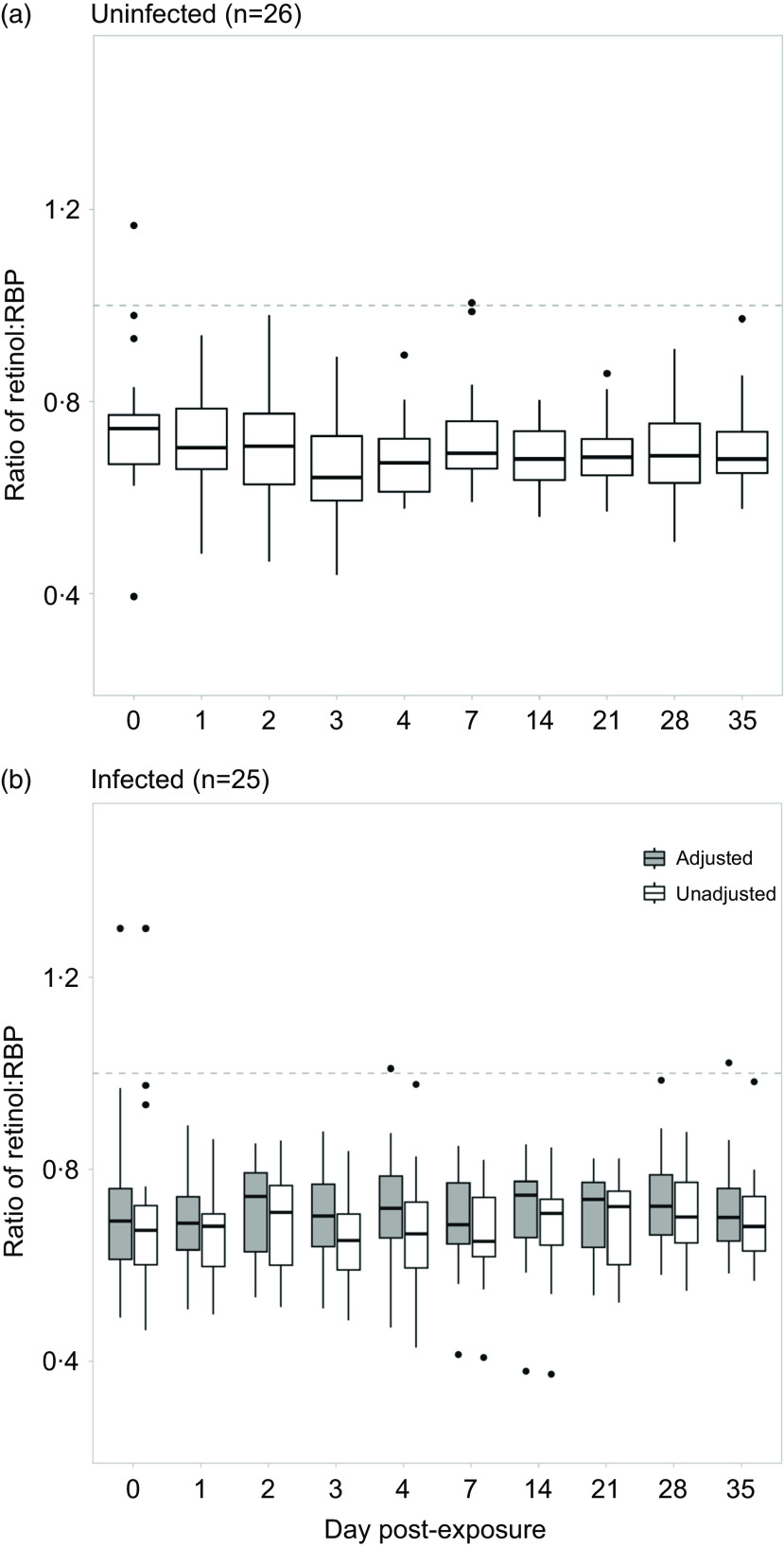
Evaluating the mean ratio between retinol and RBP among a) uninfected and b) infected individuals over the course of a norovirus challenge study. Retinol and RBP were adjusted using the BRINDA approach. A new adjusted ratio variable was created by dividing the two adjusted biomarkers.

For objective 1, the median molar ratio between retinol and RBP pooled across days among uninfected individuals was 0·68 (interquartile range = 0·12) and was significantly lower than the theoretically expected value of 1·0 (*P*-value <0·001). The median ratio among uninfected individuals within each day post-exposure ranged from 0·64–0·74 (Fig. 1(a)). Less than 0·1 % of data points of all data points (*n* 240) were between a ratio of 0·90–1·1 (+/− 10 % of 1·0). The retinol:RBP ratio was normally distributed when pooled across all days, but slightly right-skewed for days 0, 4, 7, 21, 28 and 25 post-exposure. One-sample Wilcoxon tests revealed that on each day, the ratio was significantly lower than the expected value of one (*P*-value <0·001). The median unadjusted ratio among infected individuals was 0·68 (interquartile range = 0·13) and was significantly lower than 1·0 (*P*-value<0·001). When separated by day post-exposure, the median unadjusted ratio among infected individuals ranged from 0·64 to 0·72 (Fig. 1(b)).

**Fig. 2.**
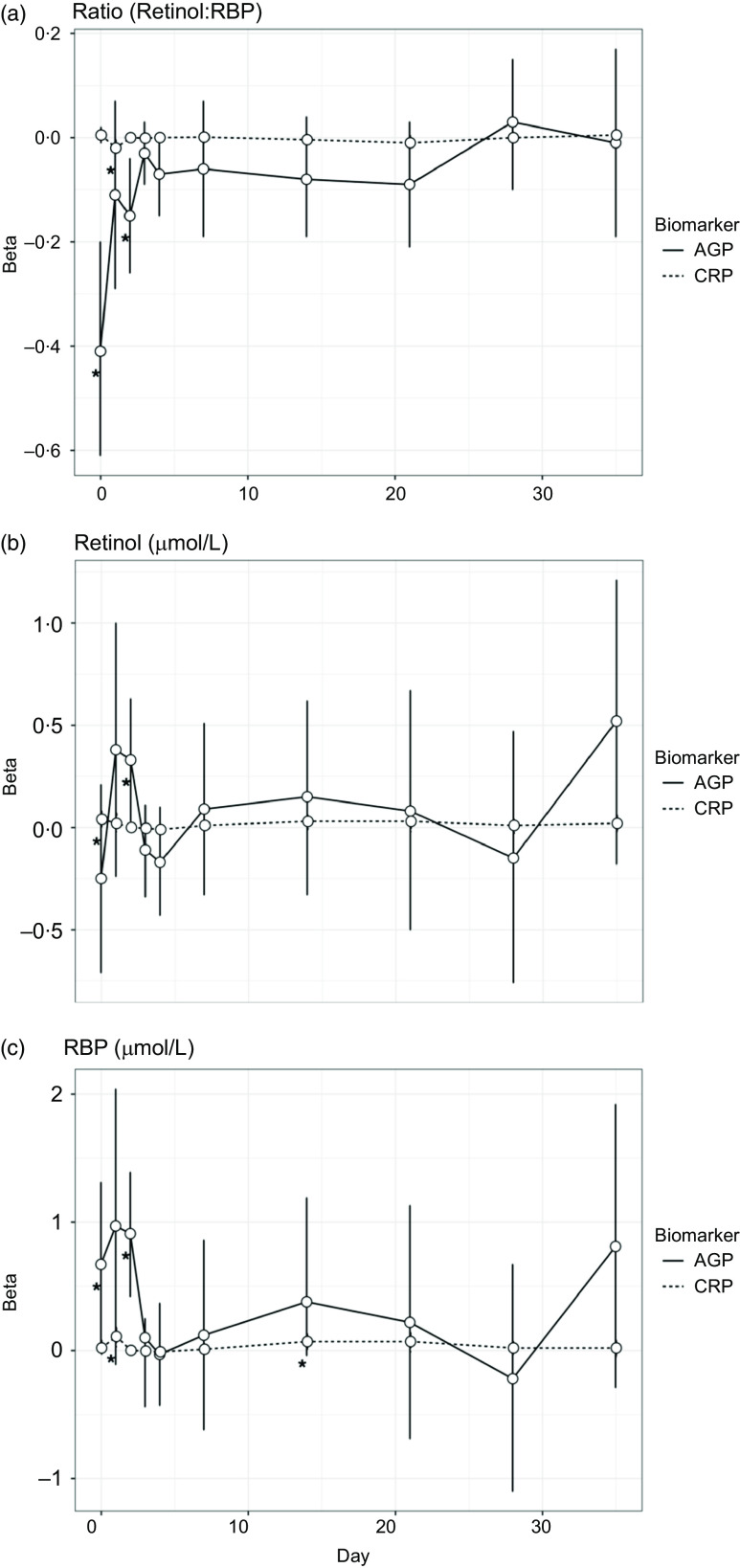
Associations between inflammatory biomarkers and vitamin A biomarkers over the course of a norovirus challenge study. All individuals (*n* 51) were included in the models for this analysis. AGP is *α*-1-acid glycoprotein (g/l), and CRP is C-reactive protein (mg/l). Betas for these figures were generated using separate linear regression models by day, to evaluate the association between each inflammatory biomarker (CRP and AGP) and the three outcomes: retinol, retinol binding protein (RBP) and the molar ratio of retinol to RBP (in three separate models).

For objective 2, the inflammatory biomarker AGP was significantly associated with the retinol:RBP ratio across all days (Table 2). This overall effect was driven by the association between AGP and the retinol:RBP ratio during the acute phase response on days 0 and 2 post-challenge (Fig. 2(a)). There was no significant overall association between CRP and the retinol:RBP ratio across all days, but CRP was significantly associated with the ratio only on day 1 post-challenge in the daily stratified models (Fig. 2(a)). We investigated whether changes in the ratio due to inflammation were driven by retinol or RBP. Across all study days, AGP was significantly associated with retinol in the unadjusted model and with RBP in the model adjusted for CRP (Table 2). In the models stratified by day, AGP was significantly positively associated with retinol on day 2 post-infection and RBP on days 0, 1 and 2 post-challenge (Fig. 2(b) and (c)). CRP was significantly associated with both retinol and RBP in both the adjusted and unadjusted models (Table 2). When stratified by day, CRP was significantly and positively associated with retinol on day 0 post-challenge, and with RBP on days 1 and 14 post-challenge (Fig. 2(b) and (c)). These effects were the same when CRP was controlled for in the AGP models and vice versa.

**Table 2.** Association between inflammatory biomarkers and retinol, RBP and the unadjusted retinol:RBP ratio among adults (*n* 51†) over 1 month following exposure to a norovirus immunologic challenge

Outcome	Predictor	*β*	se	*P*-value
Unadjusted retinol:RBP ratio	AGP‡	−0·087	0·019	<0·001*
	CRP§	−0·0004	0·001	0·53
	AGP (controlling for CRP)‖	−0·098	0·021	<0·001*
	CRP (controlling for AGP)‖	0·001	0·001	0·20
Retinol	AGP	−0·11	0·047	0·01*
	CRP	−0·007	0·001	<0·001*
	AGP (controlling for CRP)	0·009	0·054	0·86
	CRP (controlling for AGP)	−0·007	0·002	<0·001*
RBP	AGP	0·061	0·076	0·42
	CRP	−0·013	0·002	0·01*
	AGP (controlling for CRP)	0·378	0·083	<0·001*
	CRP (controlling for AGP)	−0·018	0·002	<0·001*

* Significance of the *P*-value at an alpha level of 0.05.

† One individual was excluded due to missing baseline data. There were a total of 8 missing person-time observations from the final dataset (*n* 502).

‡ AGP is *α*-1-acid glycoprotein (g/l).

§ CRP is C-reactive protein (mg/l).

‖ Mixed effects regression model with random intercept for study subject and controlling for either AGP or CRP and day post-exposure as a categorical variable.

For objective 3, given that inflammatory biomarkers were significantly associated with retinol, RBP and the ratio, we corrected the vitamin A measurements using the BRINDA adjustment method. The median inflammation-adjusted ratios among infected individuals was 0·71 (interquartile range = 0·13) and was also significantly lower than one (*P*-value<0·001). The median inflammation-adjusted ratios ranged from 0·68 to 0·74 when separated by day post-exposure (Fig. 1(b)). The unadjusted and adjusted ratio data were normally distributed when pooled across days. However, unadjusted ratio data were non-normally distributed on days 0, 4, 7, 21 and 35 post-exposure and adjusted ratio data were non-normally distributed on days 0, 2, 4, 6, 14, 21 and 35 post-exposure. Using paired Wilcoxon tests, we found that the median inflammation-adjusted ratio was higher than the median unadjusted ratio both when pooled and separated across days (*P*-value <0·001). There were fewer outliers in the unadjusted ratio measurements among infected individuals compared with uninfected individuals (*n* 6). After adjusting for inflammation, there were four outliers remaining. 0·02 % of all data points (*n* 240) were between a ratio of 0·9·0–1·1 (+/− 10 % of 1·0).

## Discussion

The objectives of this analysis were to (1) assess the molar ratio between retinol and RBP over the course of a norovirus infection; (2) assess whether inflammation may alter retinol, RBP and the expected (unadjusted) 1:1 molar ratio between these two biomarkers; and (3) understand whether the influence of inflammation could explain the deviation in the expected 1:1 molar ratio for retinol:RBP. We found that the molar ratio between retinol and RBP was significantly lower than 1:1, in norovirus-infected and uninfected individuals, across the 30 d following exposure to norovirus. The median retinol:RBP unadjusted ratio among infected and uninfected individuals was the same (0·68). Both AGP and CRP were significantly associated with retinol, RBP or the retinol:RBP ratio particularly during certain days in the acute phase response to a norovirus infection. Adjusting the vitamin A biomarkers for inflammation among infected individuals using the BRINDA adjustment approach led to a slight increase in the ratio (0·71).

The molar ratio between retinol and RBP was consistently below 1 in this direct examination of an ongoing gastroenteritis infection over time, even after adjusting for inflammation. The observed higher concentration of RBP, compared with retinol, was consistent with other study findings^(24)^. A previous study found a low molar ratio between retinol and RBP among obese adults^(25)^; data on obesity were not available from these challenge studies so we were unable to investigate this as a potential mechanism for our findings. Although it is known that inflammation can reduce serum retinol and RBP concentrations^(24,26,27)^, longitudinal data across infection are sparse. Therefore, the assumed 1:1 molar ratio of retinol to RBP, particularly in settings where the burden of inflammation-inducing infections is high, is likely inappropriate. This means that the using the cut-off for serum RBP (<0·7 μmol/l), in lieu of serum retinol (<0·7 μmol/l), to signify deficiency^(4,6)^ is also likely inappropriate. Researchers have proposed methods to adjust serum retinol and RBP concentrations for inflammation, but the utility of these adjustments need further exploration^(28)^. These results highlight the need for extra consideration of appropriate cut-offs in studies and surveys that use RBP to estimate VAD prevalence.

We assessed whether inflammation could explain why the ratio between retinol and RBP was not 1:1 in this study population and found that inflammation (both AGP and CRP) was significantly associated with vitamin A biomarkers and the ratio between them during the early days post-challenge. The serum concentrations of both retinol and RBP are impacted during the acute phase immune response to an infection^(24)^, but it is unknown whether and how inflammation impacts the molar ratio between retinol and RBP^(14)^. Differences in the associations between acute phase response proteins and retinol and RBP can be attributed to several factors. Retinol is typically transported in the serum bound to transport proteins, RBP and transthyretin; the production of both of these transport proteins is down-regulated during the acute phase response^(29)^. Researchers have also hypothesised that additional loss of serum retinol during an infection can be attributed to impaired absorption of serum retinol by the proximal renal tubules^(29)^. Overall, CRP was significantly associated with retinol and RBP, but not the ratio between the two biomarkers. AGP was significantly associated with the ratio between retinol and RBP, retinol in the unadjusted model and RBP in the adjusted model. However, we found that the relationship between AGP, CRP and vitamin A biomarkers was not consistent between retinol and RBP or over time when stratified by day. Thus, inflammation alone (as measured by AGP and CRP) cannot explain why the ratio between retinol and RBP was not 1:1 in this population. This result contributes to the evidence that RBP cut-offs may not be a good proxy for serum retinol cut-offs and thus VAD classification.

Adjusting for inflammation using the BRINDA method had little impact on the ratio between retinol and RBP. One potential explanation for the modest effect of controlling for inflammation is the relatively low levels of systemic inflammation induced by a norovirus infection in our study population^(30,31)^. Due to the low levels of inflammatory biomarkers, CRP and AGP, we were unable to apply the inflammation adjustment approach developed by Thurnham and colleagues^(15)^. Future studies of inflammation adjustment approaches may benefit from working across diseases that elicit higher levels of systemic inflammation, such as typhoid infection^(32)^, to better understand if the source or magnitude of inflammation influences vitamin A biomarkers differentially.

This work was subject to several strengths and limitations. A major strength of this research was the high-quality longitudinal data (retinol, RBP and inflammation) as part of a controlled challenge experiment. Our longitudinal analysis helps to elucidate the complicated relationship between retinol and RBP and inflammation, which is reflected in the changes observed during the acute phase response to a norovirus infection. These results provide stronger epidemiological evidence that the molar ratio between RBP and retinol is not 1:1. We were also able to assess the impact of inflammation on the ratio of retinol to RBP, a limitation of previous surveys which did not have data for both biomarkers available^(14)^. There were low levels of systemic inflammation even within the infected group, but previous analysis of this dataset demonstrated significant differences in AGP and CRP concentrations by infection group, particularly during the acute phase response^(21)^. Another limitation is the generalisability of these results to populations at the highest risk of VAD: pregnant women and children. Future longitudinal analyses of these populations could include assessment of vitamin A biomarkers and inflammatory proteins, and other inflammation adjustment methods, to better understand if RBP is an appropriate proxy for serum retinol.

### Conclusions

There is growing recognition of the importance of nutritional status among populations burdened by infectious and non-communicable diseases. Accurate assessment of population prevalence of VAD is a prerequisite to determine which public health programmes are needed, and it is likely that RBP will continue to be used for VAD assessment. The retinol to RBP ratio was significantly less than 1 among uninfected and infected individuals, irrespective of controlling for inflammation. We found that inflammatory biomarkers were individually associated with differences in serum retinol and RBP, and the ratio between the two biomarkers. The 1996 WHO guidance for assessing vitamin A status currently includes illness-related indicators, such as prevalence of diarrhoea or fever in the past 2 weeks. This analysis and results from the BRINDA project^(33)^ indicate the importance of using inflammation measurement in order to accurately assess vitamin A biomarkers. These findings support the need for WHO to provide updated guidance on a cut-off value and appropriate method for measuring and adjusting for inflammation when using RBP in population assessments of VAD.

## Supplementary material

For supplementary material accompanying this paper visit https://doi.org/10.1017/S0007114523002076. click here to view supplementary material
