# Supplementary material for: Vitamin A biomarkers were associated with α(1)-acid glycoprotein and C-reactive protein over the course of a human norovirus challenge infection
Source: Br J Nutr. 2023 Sep 11;131(3):482–8. doi: 10.1017/S0007114523002076 (PMC10784129; doi:10.1017/S0007114523002076)
Supplement: Supplementary file 1 [file S0007114523002076sup.zip › S0007114523002076sup001.docx]

Supplemental Table 1. Retinol to RBP ratio summary statistics among a population of adults exposed to a norovirus immunologic challenge, representing baseline to 35 days post-exposure

|  | **Median (IQR)** | **Wilcoxon V (P-value) ^1^** |
| --- | --- | --- |
| **Pooled^2^** |  |  |
| Uninfected | 0.68 (0.12) | - |
| Infected (unadjusted) | 0.68 (0.13) | 916 (<0.01) |
| Infected (adjusted)^3^ | 0.71 (0.13 |  |
| **Day 0** |  |  |
| Uninfected | 0.74 (0.10) | 3 (<0.01) |
| Infected (unadjusted) | 0.67 (0.12) | 10 (<0.01) |
| Infected (adjusted) | 0.67 (0.15) | 10 (<0.01) |
| **Day 1** |  |  |
| Uninfected | 0.70 (0.13) | 0 (<0.01) |
| Infected (unadjusted) | 0.68 (0.12) | 0 (<0.01) |
| Infected (adjusted) | 0.68 (0.12) | 0 (<0.01) |
| **Day 2** |  |  |
| Uninfected | 0.71 (0.15) | 0 (<0.01) |
| Infected (unadjusted) | 0.71 (0.18) | 0 (<0.01) |
| Infected (adjusted) | 0.70 (0.17) | 0 (<0.01) |
| **Day 3** |  |  |
| Uninfected | 0.64 (0.13) | 0 (<0.01) |
| Infected (unadjusted) | 0.64 (0.10) | 0 (<0.01) |
| Infected (adjusted) | 0.64 (0.10) | 0 (<0.01) |
| **Day 4** |  |  |
| Uninfected | 0.67 (0.11) | 0 (<0.01) |
| Infected (unadjusted) | 0.66 (0.14) | 0 (<0.01) |
| Infected (adjusted) | 0.66 (0.13) | 0 (<0.01) |
| **Day 7** |  |  |
| Uninfected | 0.69 (0.10) | 1 (<0.01) |
| Infected (unadjusted) | 0.64 (0.13) | 0 (<0.01) |
| Infected (adjusted) | 0.64 (0.12) | 0 (<0.01) |
| **Day 14** |  |  |
| Uninfected | 0.68 (0.10) | 0 (<0.01) |
| Infected (unadjusted) | 0.70 (0.10) | 0 (<0.01) |
| Infected (adjusted) | 0.69 (0.12) | 0 (<0.01) |
| **Day 21** |  |  |
| Uninfected | 0.68 (0.08) | 0 (<0.01) |
| Infected (unadjusted) | 0.72 (0.16) | 0 (<0.01) |
| Infected (adjusted) | 0.72 (0.15) | 0 (<0.01) |
| **Day 28** |  |  |
| Uninfected | 0.69 (0.12) | 0 (<0.01) |
| Infected (unadjusted) | 0.71 (0.13) | 0 (<0.01) |
| Infected (adjusted) | 0.71 (0.13) | 0 (<0.01) |
| **Day 35** |  |  |
| Uninfected | 0.68 (0.09) | 0 (<0.01) |
| Infected (unadjusted) | 0.68 (0.12) | 0 (<0.01) |
| Infected (adjusted) | 0.68 (0.12) | 0 (<0.01) |

^1^Pairwise Wilcoxon rank test used to test for significant differences between adjusted and unadjusted ratios among infected individuals

^2^There were 51 total participants in the study. Of these, 25 individuals became infected and 16 individuals were uninfected with norovirus.

^3^Both retinol and RBP measurements were adjusted for inflammation using the BRINDA approach
