# Supplementary material for: Vitamin A biomarkers were associated with α(1)-acid glycoprotein and C-reactive protein over the course of a human norovirus challenge infection
Source: Br J Nutr. 2023 Sep 11;131(3):482–8. doi: 10.1017/S0007114523002076 (PMC10784129; doi:10.1017/S0007114523002076)
Supplement: Supplementary file 1 [file S0007114523002076sup.zip › S0007114523002076sup002.docx]

Supplemental Table 2. Association between inflammatory biomarkers and unadjusted ratio, retinol, and RBP stratified by day^1^

|  |  | **Unadjusted retinol:RBP ratio** | | **Retinol (μmol/L)** | | **RBP (μmol/L)** | |
| --- | --- | --- | --- | --- | --- | --- | --- |
| **Day** | **Predictor** | **β**^2^ | **p-value** | **β**^2^ | **p-value** | **β**^2^ | **p-value** |
| **0** | AGP^3^ | -0.41 | <0.01* | -0.25 | 0.28 | 0.67 | 0.04* |
|  | CRP^4^ | 0.005 | 0.61 | 0.04 | 0.03* | 0.02 | 0.35 |
| **1** | AGP | -0.11 | 0.22 | 0.38 | 0.22 | 0.97 | 0.08* |
|  | CRP | -0.02 | <0.01* | 0.02 | 0.32 | 0.11 | 0.01* |
| **2** | AGP | -0.15 | 0.01* | 0.33 | 0.03* | 0.91 | 0.0004* |
|  | CRP | 0.0000001 | - | 0 | 0.96 | 0.001 | 0.94 |
| **3** | AGP | -0.03 | 0.35 | -0.11 | 0.32 | 0.1 | 0.57 |
|  | CRP | -0.001 | 0.23 | -0.004 | 0.08 | -0.004 | 0.29 |
| **4** | AGP | -0.07 | 0.07 | -0.17 | 0.22 | -0.03 | 0.88 |
|  | CRP | 0.0001 | 0.94 | -0.01 | 0.29 | -0.01 | 0.22 |
| **7** | AGP | -0.06 | 0.37 | 0.09 | 0.66 | 0.12 | 0.74 |
|  | CRP | 0.001 | 0.68 | 0.01 | 0.27 | 0.01 | 0.52 |
| **14** | AGP | -0.08 | 0.18 | 0.15 | 0.54 | 0.38 | 0.34 |
|  | CRP | -0.004 | 0.36 | 0.03 | 0.12 | 0.07 | 0.02* |
| **21** | AGP | -0.09 | 0.15 | 0.08 | 0.77 | 0.22 | 0.62 |
|  | CRP | -0.01 | 0.24 | 0.03 | 0.26 | 0.07 | 0.09 |
| **28** | AGP | 0.03 | 0.67 | -0.15 | 0.64 | -0.22 | 0.63 |
|  | CRP | -0.0001 | 0.82 | 0.01 | 0.67 | 0.02 | 0.37 |
| **35** | AGP | -0.01 | 0.93 | 0.52 | 0.14 | 0.81 | 0.15 |
|  | CRP | 0.005 | 0.38 | 0.02 | 0.38 | 0.02 | 0.65 |

^1^All individuals (n=51) were included in the models for this analysis

^2^Betas for these figures were generated using seperate linear models by day, to evaluate the association between each inflammatory biomarker (CRP and AGP) and the three outcomes: retinol, RBP, and the molar ratio of retinol to RBP (in three separate models).

^3^AGP α(1)-acid glycoprotein (g/L)

^4^C-reactive protein (mg/L)
